# Deep Segmentation Feature-Based Radiomics Improves Recurrence Prediction of Hepatocellular Carcinoma

**DOI:** 10.34133/2022/9793716

**Published:** 2022-04-04

**Authors:** Jifei Wang, Dasheng Wu, Meili Sun, Zhenpeng Peng, Yingyu Lin, Hongxin Lin, Jiazhao Chen, Tingyu Long, Zi-Ping Li, Chuanmiao Xie, Bingsheng Huang, Shi-Ting Feng

**Affiliations:** ^1^Department of Radiology, The First Affiliated Hospital, Sun Yat-sen University, Guangzhou, China; ^2^Medical AI Lab, School of Biomedical Engineering, Health Science Centre, Shenzhen University, Shenzhen, China; ^3^Shenzhen-Hong Kong Institute of Brain Science-Shenzhen Fundamental Research Institutions, ShenzhenChina; ^4^Department of Medical Imaging and Interventional Radiology, Sun Yat-sen University Cancer Center, State Key Laboratory of Oncology in South China, Collaborative Innovation Center for Cancer Medicine, Guangzhou, China

## Abstract

*Objective and Impact Statement*. This study developed and validated a deep semantic segmentation feature-based radiomics (DSFR) model based on preoperative contrast-enhanced computed tomography (CECT) combined with clinical information to predict early recurrence (ER) of single hepatocellular carcinoma (HCC) after curative resection. ER prediction is of great significance to the therapeutic decision-making and surveillance strategy of HCC. *Introduction*. ER prediction is important for HCC. However, it cannot currently be adequately determined. *Methods*. Totally, 208 patients with single HCC after curative resection were retrospectively recruited into a model-development cohort (n=180) and an independent validation cohort (n=28). DSFR models based on different CT phases were developed. The optimal DSFR model was incorporated with clinical information to establish a DSFR-C model. An integrated nomogram based on the Cox regression was established. The DSFR signature was used to stratify high- and low-risk ER groups. *Results*. A portal phase-based DSFR model was selected as the optimal model (area under receiver operating characteristic curve (AUC): development cohort, 0.740; validation cohort, 0.717). The DSFR-C model achieved AUCs of 0.782 and 0.744 in the development and validation cohorts, respectively. In the development and validation cohorts, the integrated nomogram achieved C-index of 0.748 and 0.741 and time-dependent AUCs of 0.823 and 0.822, respectively, for recurrence-free survival (RFS) prediction. The RFS difference between the risk groups was statistically significant (P<0.0001 and P=0.045 in the development and validation cohorts, respectively). *Conclusion*. CECT-based DSFR can predict ER in single HCC after curative resection, and its combination with clinical information further improved the performance for ER prediction.

## 1. Introduction

Hepatocellular carcinoma (HCC) is one of the most common cancers and the leading cause of cancer-related death worldwide, especially in East Asia [1]. Given the limitation of organ shortage for liver transplantation, hepatic resection is the main treatment option for patients with single HCC with well-preserved liver function, as multifocality is associated with a higher recurrence rate and impaired survival [2, 3]. Patients with early-stage HCC have a favorable prognosis after resection, with a 5-year survival of 71.1% to 77.2% [4, 5]. However, early-stage HCC resection is still associated with a 3-year recurrence rate of 40.1% to 43.3%, which is the main factor contributing to the poor outcome of patients with HCC [4, 5]. Compared with late recurrence, early recurrence (ER, <2 years) after resection is mainly related to the characteristics of the tumor, such as microscopic vascular invasion (MVI) and surgical factors, which account for more than 70% of the recurrence of HCC [6, 7]. Although there is currently no widely accepted treatment to reduce the recurrence of HCC after resection [8], accurate prediction of ER before resection is of great significance to the grouping of clinical trials, therapeutic decision-making, and surveillance strategy of HCC, especially for early-stage single HCC patients [9, 10]. Unfortunately, the recurrence of single HCC cannot currently be adequately determined preoperatively.

Studies have reported some important histopathologic factors such as tumor differentiation, MVI, and microsatellite nodules for ER prediction after HCC resection [8, 11]. Genetic or molecular signatures have also been investigated for predicting HCC recurrence [2, 12]. Nevertheless, these factors and signatures are obtained through invasive surgery or biopsy, and controversy still exists regarding the prediction performance of these signatures [12]. Moreover, because of the spatial and temporal heterogeneous nature of tumors, genetic or molecular signatures identified from small local tissue do not allow for real-time and comprehensive characterization of the tumors [12, 13].

As a noninvasive method, medical imaging has been widely used in the surveillance, diagnosis, staging, and prognosis of HCC [8]. Recently, radiomics has become a rapidly developing machine learning-based image analysis method: by extracting high-throughput quantitative features from images, data can be mined and analyzed to retrieve valuable diagnostic and prognostic information for clinical decision-making [14, 15]. Several studies have applied radiomics to predict HCC recurrence and have shown promising outcomes [9, 16]; however, the traditional radiomics approach includes segmentation, feature extraction, and modeling, which is time-consuming and labor-intensive, and feature extraction is limited by the human-defined nature, which may not be adequately representative [14, 17]. Deep learning is another subtype of machine learning based on a neural network structure which extracts and learns the abstract features directly in a data-driven manner [18]. A few studies have applied deep learning in medical image analysis to predict the recurrence of HCC [19–21]. Wang et al. [19] adopted the ResNet network based on computed tomography to predict ER after HCC resection. However, their study included the patients of intermediate and advanced-stage HCC for whom resection is not recommended as the first-line treatment. Deep learning strategy for HCC recurrence risk assessment after liver transplantation based on magnetic resonance imaging [20], or after radiofrequency ablation and surgical resection based on contrast-enhanced ultrasound [21], has been reported and shown hopeful results. However, external validation was not performed in all these studies.

Huang et al. [22] recently proposed a novel and effective deep semantic segmentation feature-based radiomics (DSFR) method, which uses the segmentation network to automatically extract effective features. This method improves tumor characterization and proposes a feature selection module to achieve effective information integration. This novel approach overcomes the shortcomings of deep learning- (DL-) based classification networks that struggle to capture representative features of lesion regions and are easy to overfit and the shortcomings of traditional radiomics mentioned above. Huang et al. demonstrated that the proposed DSFR method consistently outperforms DL and radiomics in different tasks [22].

In this study, we aimed to develop and validate a DSFR model based on preoperative contrast-enhanced computed tomography (CECT) combined with clinical information to predict ER (<2 years) of single HCC after curative resection. We hypothesized that the features automatically extracted by the DL segmentation network would be effective for ER prediction. Since studies showed that clinical information complements radiomics features in predictive models [9, 23], we further hypothesized that the semantic segmentation radiomics features combined with clinical information would improve the performance of ER prediction of HCC.

## 2. Results

### 2.1. Baseline Characteristics

Detailed baseline characteristics of all 208 patients are shown in Table 1. There was no significant difference in the recurrence rate and median recurrence-free survival (RFS) between the development and validation cohorts (median RFS: development cohort, 14.8 months; validation cohort, 17.7 months, P=0.978). The demographic, laboratory parameters, and visual analysis features had no significant difference between the two cohorts (P>0.05).

**Table 1:** Baseline characteristics of patients in different cohorts.

Variable	Development cohort (n=180)	Validation cohort (n=20)	Statistical test	P value
Patient demographics				
Gender, n (%)			Pearson’s chi-square test	0.293
Male	146 (81.1)	25 (89.3)		
Female	34 (18.9)	3 (10.7)		
Age, years, mean (SD)	40.7 (5.1)	51.9 (13.6)	Student’s t-test	0.947
Laboratory parameters				
HBsAg, n (%)			Pearson’s chi-square test	0.100
Negative	19 (10.6)	6 (21.4)		
Positive	161 (89.4)	22 (78.6)		
HBV-DNA, IU/*μ*L, n (%)			Pearson’s chi-square test	0.925
<100	53 (29.4)	8 (28.6)		
≥100	127 (70.6)	20 (71.4)		
AFP, *μ*g/L, n (%)			Pearson’s chi-square test	0.627
<400	107 (59.4)	18 (64.3)		
≥400	73 (40.6)	10 (25.7)		
Child grade, n (%)			Fisher’s exact probability test	0.665
A	176 (97.8)	27 (96.4)		
B	4 (2.2)	1 (3.6)		
TB, *μ*mol/L, median (IQR)	13.7 (10.8, 17.0)	13.0 (9.7, 16.9)	Mann–Whitney U test	0.424
ALB, g/L, n (%)				0.090
≥35	163 (90.6)	28 (100.0)		
<35	17 (9.4)	0 (0.0)		
ALT, U/L, median (IQR)	33.0 (23.0, 51.0)	35.9 (25.0, 48.4)	Mann–Whitney U test	0.988
GGT, U/L, median (IQR)	49.0 (29.0, 87.8)	60.3 (29.3, 133.1)	Mann–Whitney U test	0.333
Visual features				
Tumor diameter, mm, median (IQR)	47.0 (32.3, 68.0)	46.5 (35.8, 88.3)	Mann–Whitney U test	0.688
Attenuation of tumor on nonenhanced CT, n (%)			Pearson’s chi-square test	0.746
Homogeneous	65 (36.1)	11 (39.3)		
Nonhomogeneous	115 (63.9)	17 (60.7)		
Vessels in tumor, n (%)			Pearson’s chi-square test	0.077
Absent	71 (39.4)	16 (57.1)		
Present	109 (60.6)	12 (42.9)		
Irregular rim-like enhancement, n (%)			Pearson’s chi-square test	0.952
Absent	147 (81.7)	23 (82.1)		
Present	33 (18.3)	5 (17.9)		
Capsule appearance, n (%)			Pearson’s chi-square test	0.423
Incomplete	150 (83.3)	25 (89.3)		
Complete	30 (16.7)	3 (10.7)		
Tumor margin, n (%)			Pearson’s chi-square test	0.381
Smooth	127 (70.6)	22 (78.6)		
Nonsmooth	53 (29.4)	6 (21.4)		
Peritumoral arterial enhancement, n (%)			Pearson’s chi-square test	0.637
Absent	154 (85.6)	23 (82.1)		
Present	26 (14.4)	5 (17.9)		
Cirrhosis, n (%)			Pearson’s chi-square test	0.235
Absent	136 (75.6)	24 (85.7)		
Present	44 (24.4)	4 (14.3)		
Clinical outcome				
No. of recurrences, n (%)	114 (63.3)	18 (64.3)	Pearson’s chi-square test	0.922
Median RFS, months (95% CI)	14.8 (9.5, 20.1)	17.7 (0.6, 34.8)	Log-rank test	0.978

SD: standard deviation; HBsAg: hepatitis B surface antigen; HBV: hepatitis B virus; AFP: alpha-fetoprotein; TB: total bilirubin; ALB: albumin; ALT: alanine aminotransferase; GGT: gamma-glutamyl transferase; IQR: interquartile range; CT: computed tomography; RFS: recurrence-free survival.

### 2.2. Prediction Performance of DSFR, DSFR-C Models, and the Model by Visual Features

**Figure 1:**
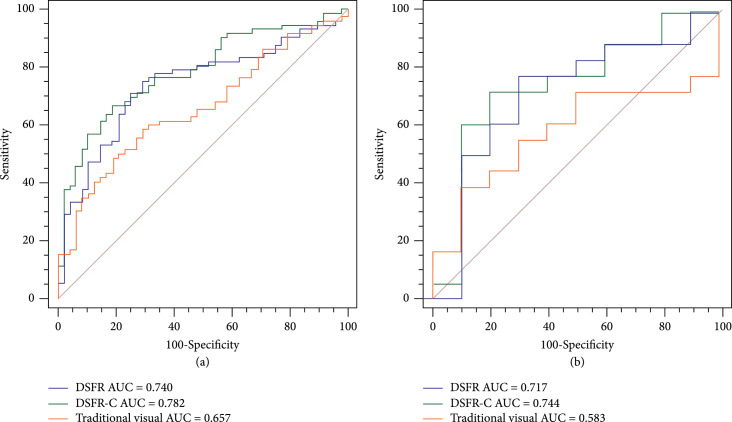
Comparison of ROC curves among different models for predicting ER of HCC ((a) development cohort; (b) validation cohort). ROC: receiver operating characteristic; ER: early recurrence; HCC: hepatocellular carcinoma; DSFR: deep semantic segmentation feature-based radiomics; DSFR-C: deep semantic segmentation feature-based radiomics with clinical information.

The Dice similarity coefficient (DSC) of the segmentation model was 0.640 for the model based on arterial phase (AP) and 0.717 for the model based on portal phase (PP). The performances of DSFR models based on AP, PP, and dual-phase (DP, using both AP and PP images) were compared, and that based on PP showed the best performance (area under receiver operating characteristic curve (AUC): development cohort, 0.740; validation cohort, 0.717) (Table 2, Figure 1) which was selected as the final DSFR model. The details of the prediction performance of DSFR based on different phases are presented in Supplementary Materials (section 3 of Supplementary Materials). Then, the DSFR-C model incorporating deep features of the DSFR model based on PP and clinical information was established. In the development cohort, the DSFR-C model achieved the highest AUC of 0.782, while the DSFR model and model by visual features only achieved AUCs of 0.740 and 0.657, respectively. In the independent validation cohort, AUCs decreased slightly in the performance to predict ER of HCC and were consistent with the performance of the development cohort. The detailed results are summarized in Table 2 (Figure 1).

**Table 2:** The performances of different models for the prediction of ER of HCC.

	Development cohort					Validation cohort				
	AUC (95% CI)	ACC	SEN	SPEC	P value${}^{*}$	AUC (95% CI)	ACC	SEN	SPEC	P value${}^{*}$
DSFR	0.740 (0.652, 0.816)	0.733	0.750	0.708	Ref	0.717 (0.516, 0.869)	0.750	0.778	0.700	Ref
DSFR-C	0.782 (0.698, 0.853)	0.725	0.667	0.812	0.042	0.744 (0.545, 0.889)	0.750	0.722	0.800	0.028
Model with visual features	0.657 (0.565, 0.742)	0.617	0.486	0.813	0.149	0.583 (0.383, 0.765)	0.572	0.389	0.900	0.287

${}^{*}$DeLong’s test. ER: early recurrence; HCC: hepatocellular carcinoma; AUC: area under the curve; ACC: accuracy; SEN: sensitivity; SPEC: specificity; DSFR: deep semantic segmentation feature-based radiomics; Ref: reference; DSFR-C: deep semantic segmentation feature-based radiomics with clinical information.

### 2.3. Visualization of Deep Segmentation Features of the DSFR Model

**Figure 2:**
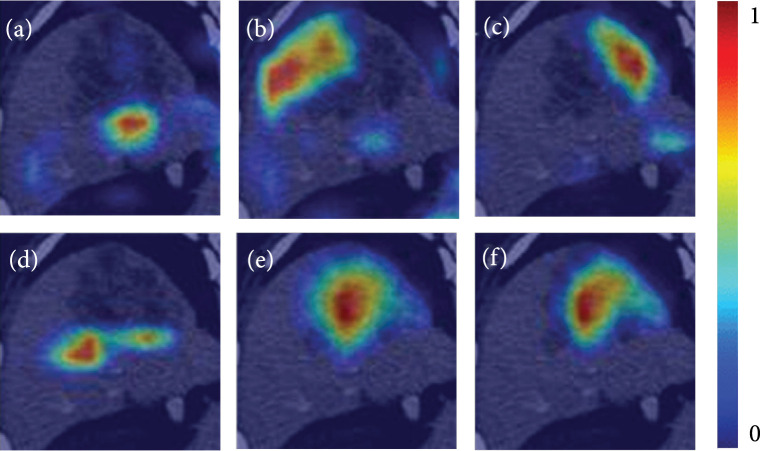
Example of visualization of the deep segmentation features of the DSFR model. (a–f) Saliency maps of the top six features with the highest weights in the DSFR model. The highlighted red and blue areas represent the regions where the deep segmentation features are extracted, while the red regions indicated higher weights for prediction. As shown, the top six features with the highest weights in the DSFR model focused on (a, d, c) the peritumoral area, (e) tumor region, (b, f) or both, respectively. Such focused regions contained information significantly associated with recurrence, such as tumor size, peripheral enhancement, nonsmooth margin, and capsule appearance. DSFR: deep semantic segmentation feature-based radiomics.

To improve the understanding of the functional mechanism of the DSFR model and to verify our hypothesis, we created visualized deep feature maps. We found that the top six features with the highest weights in the DSFR model were all focused on the peritumoral area, tumor region, or both (Figure 2).

### 2.4. RFS Prediction by DSFR Signature and Integrated Nomogram

**Figure 3:**
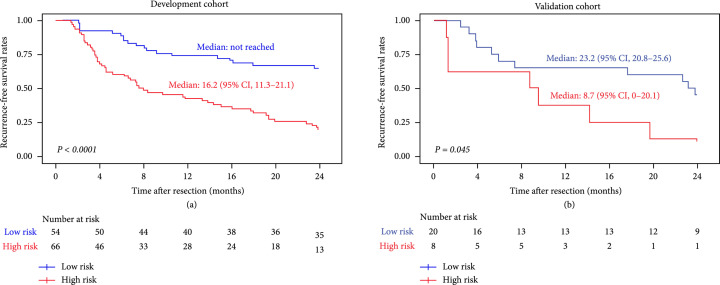
The Kaplan-Meier survival curves of RFS in the (a) development and (b) validation cohorts. RFS: recurrence-free survival.

The Kaplan-Meier curves showed a significant difference in RFS between low-risk and high-risk subgroups in the development and validation cohorts. The median RFS of the low-risk subgroup was significantly longer than that of the high-risk subgroup in both cohorts (P<0.05) (Figure 3).

**Figure 4:**
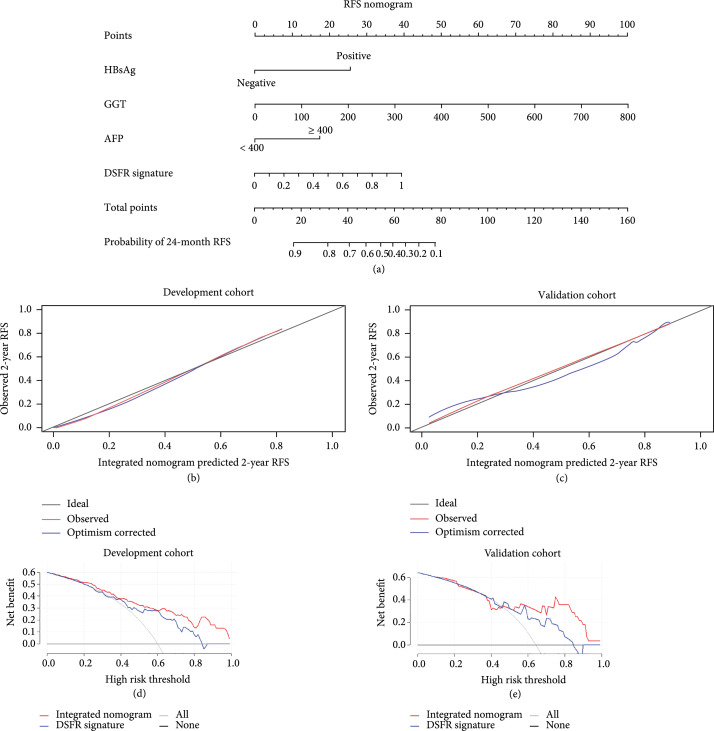
(a) Nomogram to evaluate individualized 24-month RFS for single HCC after curative resection, (b, c) along with calibration, (d, e) and decision curves (d, e). RFS: recurrence-free survival; HCC: hepatocellular carcinoma; AUC: area under the curve; HBsAg: hepatitis B surface antigen; AFP: alpha-fetoprotein; GGT: gamma-glutamyl transferase; DSFR: deep semantic segmentation feature-based radiomics.

Six variables, including DSFR signature-predicted ER according to the univariable Cox regression analysis (Table S1). The multivariable Cox regression analysis identified DSFR signature (P<0.0001), HBsAg (P=0.013), AFP (P=0.001), and GGT (P<0.0001) as independent predictors for ER of HCC, and the DSFR signature was the highest weighted parameter (Table 3). Based on these predictors, an integrated preoperative predictive model for ER of HCC was developed and presented as a nomogram (Figure 4(a)).

**Table 3:** Multivariable Cox regression analysis of predictors of ER in the development cohort.

	Hazard ratio (95% CI)	β (95% CI)	P value
DSFR signature	7.283 (3.207, 16.539)	1.985 (1.165, 2.806)	<0.0001
HBsAg			
Negative	Ref	Ref	
Positive	3.647 (1.310, 10.149)	1.294 (0.270, 2.317)	0.013
AFP, *μ*g/L			
<400	Ref	Ref	
≥400	2.359 (1.455, 3.825)	0.858 (0.375, 1.342)	0.001
GGT	1.006 (1.004, 1.009)	0.006 (0.004, 0.009)	<0.0001

ER: early recurrence; CI: confidence interval; DSFR: deep semantic segmentation feature-based radiomics; HBsAg: hepatitis B surface antigen; Ref: reference; AFP: alpha-fetoprotein; GGT: gamma-glutamyl transferase.

The C-index of the integrated nomogram for ER prediction of HCC in the development and validation cohorts was 0.748 (95% confidence interval (CI): 0.72, 0.82) and 0.741 (95% CI: 0.72, 0.84), respectively. The predictive performance of the integrated nomogram was superior to that of using DSFR signature alone, in both study cohorts (C-index in the development cohort: 0.664, validation cohort: 0.630) (Table 4). Using a time-dependent ROC analysis, we found that the integrated nomogram achieved time-dependent AUC (tdAUC) of 0.822 for 2-year RFS prediction in the validation cohort and improved the prediction of HCC recurrence compared with the DSFR signature model at various time points, in both study cohorts (Figure S1). Complete details of the tdAUC for each model are reported in Table 4. The calibration curves of the nomogram demonstrated good agreement between the prediction results and the observations in both cohorts (Figures 4(b) and 4(c)). The decision curve analysis graphically demonstrated that the integrated nomogram provided more net benefits across the range of reasonable threshold probabilities compared with the Cox regression model using the DSFR signature alone, in both study cohorts (Figures 4(d) and 4(e)).

**Table 4:** Prognostic performance of integrated nomogram compared with that using deep learning signature alone.

		Integrated nomogram (95% CI)	P value	DSFR signature (95% CI)	P value
Harrell’ s C-index	Development cohort	0.748 (0.691, 0.805)	Ref	0.664 (0.604, 0.726)	<0.001
	Validation cohort	0.741 (0.669, 0.813)	Ref	0.630 (0.504, 0.755)	0.015
tdAUC (2 years)	Development cohort	0.823 (0.750, 0.895)	Ref	0.742 (0.651, 0.833)	0.019
	Validation cohort	0.822 (0.664, 0.981)	Ref	0.717 (0.504, 0.930)	0.328

CI: confidence interval; Ref: reference; tdAUC: time-dependent area under the curve.

## 3. Discussion

We conducted a dual-center study to develop and validate a DSFR model to predict ER of single HCC after curative resection. This method showed better performance than the model based on traditional visual features, especially in combination with clinical information. Survival analysis showed that the established DSFR signature was an independent risk factor and could accurately stratify the ER risk of patients with a single HCC. The nomogram-integrating DSFR signature and clinical information could accurately predict ER of single HCC after resection and showed superior predictive performances to the model with DSFR signature alone.

The recently proposed DSFR method [22] has been designed to automatically extract representative features from the segmentation model, focusing on the target and characterizing the tumor region. To improve our grasp on the mechanism of the DSFR model to predict ER and validate our hypothesis, we created visualized deep feature maps using the Grad-CAM method [24]. The saliency maps showed that the six features with the top highest weights in DSFR model building were all derived from the peritumoral area, tumor region, or both. Studies have shown that peritumoral changes, as well as tumor features, of HCC include massive information and play an important role in predicting HCC recurrence, either in traditional radiomics [16, 25] or traditional visual features analysis [26, 27]. Therefore, we suppose that the features extracted by the segmentation network in the DSFR model represent the characteristics of peritumoral area and tumor region, and these features contain effective information for predicting early recurrence of HCC. Compared with the traditional radiomics approach, the DSFR method alleviates the time-consuming and labor-intensive workload regarding lesion segmentation and automatically extracts the effective features. Our study also showed that the predictive performance of the DSFR model was superior to the model by visual features, though the difference was not statistically significant. Compared with the visual features, the DSFR method extracts deep features that are less affected by experience and fatigue. All these reasons may explain the more powerful predicting performance of deep segmentation features in our study.

The results of this study showed that the DSFR model based on AP had unsatisfactory prediction performance (with an AUC of 0.646 in the development cohort). This may be affected by the poor segmentation performance based on AP images, with an average DSC of 0.640, which was lower than that of PP (0.717). A previous study which utilized DL for HCC lesion segmentation based on CT also showed that the segmentation performance of the model based on PP was better than that based on AP [28]. The poor segmentation performance based on AP may be due to certain features of tumoral margins in AP (such as the peritumoral arterial enhancement) leading to an unclear outline of the lesions, compared to that in PP in which the lesions presented as washout. In addition, in this study, the scan time of the AP and PP adopted a fixed delay time; thus, some patients may have insufficient enhancement in the AP scan with fixed delay time.

The fusion of the dual-phase features generated worse results than the one based on PP. We conducted the correlation analysis to attempt to explain this (shown in section 3 of Supplementary Materials, Table S3 and Table S4). The results showed that the features from the two phases cannot be considered completely complementary or substantially redundant. Thus, we considered that the unsatisfactory performance of the dual-phase DSFR model may be due to the unrepresentative features from AP. Another reason may be the simple strategy of feature fusion used in our study, which may not fully utilize the complementary information within these two phases.

Wang et al. [19] applied the ResNet classification network based on CECT to predict ER (<1 year) after HCC resection and achieved an AUC of 0.723. However, the study included the patients of BCLC stages B and C, for which resection is not recommended as the first-line treatment, and patients have different prognoses. External validation was not performed either [19]. Our study not only achieved higher performance by using the DSFR method but also selected patients with BCLC stage 0-A single HCC, for whom the ER prediction is more important in the choice of treatment strategies.

Our study showed that the DSFR signature could accurately stratify the patients with different risks of ER in both the development and validation cohorts. The multivariate Cox regression analysis demonstrated that the DSFR signature was an independent risk factor for ER and made the largest contribution to prediction in the Cox proportional hazard model. Moreover, the results of our study showed that incorporating DSFR signature and clinical information could further improve the predictive performance for ER, both in the DSFR method and nomogram. The nomogram model integrating the DSFR signature and clinical information improved the 2-year RFS prediction tdAUC at various time points in both study cohorts, compared with using the DSFR signature alone. The decision curve analysis also demonstrated that the integrated nomogram provided more net benefit. Similar results have been reported in the literature [16, 23, 29]. The reason may be that laboratory tests can provide additional predictive information independent of imaging, such as the function of tumors to secrete AFP, liver function, or tumor function information reflected by GGT.

This study has some limitations. First, it was a retrospective study, and there might be bias in patient’s inclusion. However, we showed that there was no significant difference in the baseline characteristics between the two cohorts, and external validation was conducted to justify the robustness of the model. Second, the sample size was relatively small, and studies with a larger sample size remain necessary to further validate the performance of our model in the future. Third, the prediction performance (AUC) of the DSFR model based on AP was unsatisfactory. Improving the segmentation accuracy of the AP images and combining the AP and PP features may further improve the prediction performance.

In conclusion, our study showed that radiomics features based on the deep segmentation network and CECT can predict ER in single HCC after curative resection. The DSFR signature was an independent risk factor of ER and could accurately stratify the patients for ER risk with single HCC. The combination of semantic segmentation radiomics features with clinical information further improved the performance of ER prediction of HCC.

## 4. Materials and Methods

We first developed DSFR models based on CECT and clinical information for ER prediction in single HCC patients after curative resection. We subsequently conducted a multivariate Cox regression analysis and built nomograms by incorporating the DSFR signature, clinical information, and survival data. Both the DSFR and Cox regression models were validated in an independent cohort.

**Figure 5:**
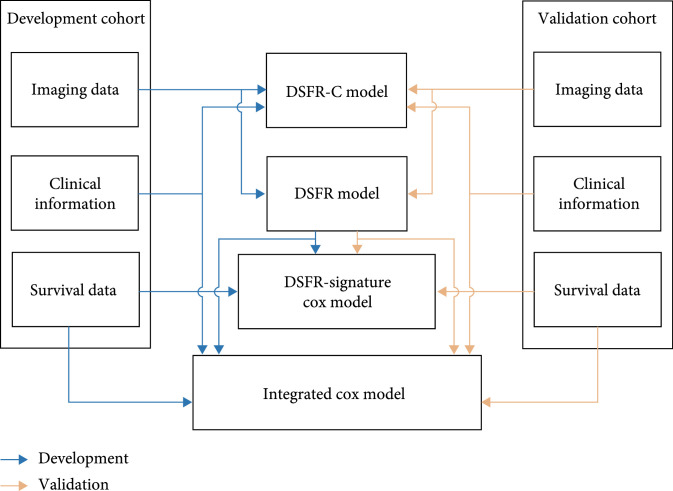
Flow diagram of study design. DSFR-C: deep semantic segmentation feature-based radiomics with clinical information; DSFR: deep semantic segmentation feature-based radiomics.

The institutional review boards of both participating institutions (Institution 1, the First Affiliated Hospital of Sun Yat-sen University, Guangzhou, China; Institution 2, Sun Yat-sen University Cancer Center, Guangzhou, China) approved this retrospective study, and written informed consent was obtained from all patients in the study. The study was conducted in accordance with the Declaration of Helsinki. Figure 5 shows the overall scheme of our study.

### 4.1. Patients

Consecutive adult patients (≥18 years) after curative resection for HCC were confirmed pathologically and recruited in the study. The inclusion criteria were (1) preoperative CT or MRI showing a single HCC without satellite nodules or infiltrative HCCs with obscure borders, (2) no macrovascular invasions or extrahepatic metastasis, (3) preoperative CECT within 1 month before surgery, (4) no pretreatment history before resection (including liver transplantation, ablation, TACE, radiotherapy, or chemotherapy), and (5) a Child-Pugh grade of A or B, and a performance status score of 0 or 1. These criteria met the Barcelona clinical liver cancer (BCLC) stage 0 or A [11]. Patients were excluded from the study if (1) there were artifacts in the preoperative CT images, (2) postoperative imaging showed only extrahepatic metastasis without intrahepatic recurrence, (3) comorbidity with other primary malignancies was present, (4) the HCCs ruptured before surgery, or (5) imaging, clinical, or follow-up data were incomplete or not available. Figure S2 shows the patient recruitment workflow.

Overall, 208 patients were enrolled in this study from two tertiary hospitals, with 180 recruited between January 2011 and December 2016 from Institution 1 used to develop and cross-validate the models of preoperative prediction of ER. Twenty-eight patients, recruited between January 2014 and December 2016 from Institution 2, were used to externally validate these models. Student’s t-test and Mann–Whitney’s U test were used for continuous variables; $\chi^{2}$ or Fisher’s exact tests were used for categorical variables to evaluate the difference between the development and validation cohorts.

### 4.2. CT Scanning, Clinical Information, and Recurrence

Preoperative CECT scans were performed, and both the arterial phase (AP) and portal phase (PP) were obtained. CT scanners from two manufacturers (Canon Medical System, Otawara, Japan, for Institution 1; and Philips Medical Systems, Best, Netherlands, for Institution 2) were used. All CT scans were performed with a tube voltage of 120 kVp. For the tube current, an automatic tube current modulation technique or fixed 250 mAs was used. The enhanced scanning time of both the AP and PP adopted a fixed delay time. The reconstruction slice thickness was 1 mm for both scanners. The details of the CT scan parameters of the two institutions are presented in Table S2. For the DSFR model development, CT images were set with a window level and width of 70 HU and 150 HU for AP, and 100 HU and 200 HU for PP, respectively.

The candidate demographic and laboratory parameters were retrieved from the clinical database, including age, sex, hepatitis B surface antigen (HBsAg) level (negative or positive), hepatitis B virus- (HBV-) DNA (IU/*μ*L, <100 or ≥100), alpha-fetoprotein (AFP) level (ng/mL, <400 or ≥400), serum total bilirubin (TB, *μ*mol/L), serum albumin (ALB, g/L, <35; ≥35), serum alanine aminotransferase (ALT, U/L), gamma-glutamyl transpeptidase (GGT, U/L), and Child-Pugh grade. The statistical analysis of all these parameters adopted the same method mentioned above.

CECT or contrast-enhanced MRI, serum AFP level, and liver function tests were performed every 3-6 months for two years after surgery. The observation endpoint was HCC intrahepatic recurrence, which was determined by CECT or MRI. The cases without recurrence were followed up for at least two years. Recurrence-free survival (RFS) was defined as the interval between the date of resection and the date of detection of the recurrence on imaging. The cases without recurrence within 2 years after surgery were censored. RFS<2 years was defined as ER [6].

### 4.3. Development and Validation of DSFR and DSFR-C Models

In this study, we built a deep learning-based segmentation model of HCC to generate high-dimensional semantic features from preoperative CECT. AP and PP images of CECT were downloaded in a Digital Imaging and Communications in Medicine format. The CT images of all patients were input into the ITK-SNAP software (version 3.6, http://www.itksnap.org). Sixty cases were randomly extracted from the 180 patients from Institution 1 and were used to train a deep learning-based segmentation model of the U-Net network. Manual segmentation of tumor lesions in preoperative AP and PP images of the extracted cases was performed by two radiologists with 10 years of experience, confirmed by a radiologist with 21 years of experience, and used as the ground truth for training the segmentation model. For training and testing of the deep learning model, images were processed in the same format as the input of the segmentation network. First, a fixed window center and window width were applied for the original CT images. Then normalization was performed. Finally, the processed images were resized to 256×256.

The Dice similarity coefficient (DSC) was adopted to select and obtain the optimal segmentation model, which was then implemented to extract the semantic segmentation features. Due to different tumor sizes, the number of semantic features generated from each case varied. Therefore, the average value of the features from the same case was calculated, which was regarded as the final segmentation feature of the case. The architecture of the segmentation network is shown in Figure S3. Details of deep learning-based segmentation model construction and deep feature extraction are provided in supplementary materials.

All patients were grouped to ER or non-ER, if they encountered recurrence or not within two years, respectively. ER prediction models were built based on those extracted deep semantic features (DSFR models). Specifically, logistic regression was chosen as the classifier to build the prediction model. The parameters of the classifier were determined by the grid search strategy, and the range of grid optimization parameters was as follows: (1) regularization strength: 0.01, 0.05, 0.1, 0.3, 0.4, 0.5, 0.6, 0.8, 1.0, and 1.2; (2) maximum number of iterations: 100, 150, 200, 400, and 500; and (3) conditions for stopping iteration optimization: 1E-5, 1E-4, 1E-3, and 1E-2. Cross-validation was adopted to search for the best hyperparameters setting. In this study, DSFR models based on AP, PP, and dual-phase (DP, using both AP and PP images) images were developed and validated in internal cross-validation and the independent external cohort and compared to obtain the one with the best performance, which was defined as the final DSFR model.

Visualization of the deep semantic segmentation features of the best model was performed by using the Grad-CAM method [24] to interpret how the DSFR model worked for ER prediction. We chose the top six features with higher weights in the classifier. Then, according to the distribution of the gradient in the segmentation model, we generated a heatmap for each selected feature. The red region in the heatmap indicated the high gradient and illustrated the significant elements in the image for the selected feature. Through the heatmaps, what the feature pays attention to was observed in the image.

Clinical information was further added to establish an integrated model (DSFR-C model), to test whether the combination of clinical information with deep segmentation features could improve the prediction performance of ER in HCC. Similarly, logistic regression was chosen as the classifier, and strategies were employed to train the model, including grid search for the hyperparameters setting and cross-validation.

Receiver operating characteristic (ROC) curves were drawn, and the performances of the ER prediction models were evaluated by the area under the curve (AUC) value, sensitivity, specificity, and accuracy in the development and independent validation cohorts. The difference between AUCs among different models was compared by DeLong’s test.

### 4.4. Development and Validation of the Model with the Imaging Features by Visual Analysis

The preoperative CT images were visually interpreted by two radiologists with more than 10 years of experience, to evaluate the following traditional visual imaging features (Figure S4): (1) the size of the tumor; (2) attenuation of the tumor on nonenhanced CT images (heterogeneous or nonheterogeneous); (3) vessels in the tumor (absent or present); (4) peritumoral arterial enhancement (absent or present); (5) irregular rim-like enhancement in the AP (absent or present); (6) the tumor margin (smooth or nonsmooth); (7) capsule appearance (complete or incomplete); and (8) cirrhosis manifestations (absent or present). Both radiologists were blind to the recurrence outcome and clinical information of the patients from both cohorts. Disagreements between the readers were discussed to obtain a final consensus and minimize interpretation bias.

An ER prediction model based on the traditional visual features was developed (the model by visual features). Logistic regression was chosen as the classifier to train the model, based on the eight traditional visual features. Then, the grid search strategy with the same range of grid optimization parameters was employed as above. The model with the best performance was subsequently selected for comparison with DSFR and DSFR-C models. AUC, sensitivity, specificity, and accuracy were also evaluated. The difference between AUCs among different models was also evaluated by DeLong’s test.

### 4.5. Survival Analysis and Nomogram Development for RFS Prediction

Both the development and validation cohorts were stratified into low-risk and high-risk subgroups, in accordance with the prediction results of non-ER or ER by the DSFR model with best performance. RFS was assessed by the Kaplan-Meier method, and differences in survival distributions between the stratified subgroups were compared by using log-rank tests.

The probability value predicted by the DSFR model with the best performance for ER prediction was defined as the DSFR signature. The clinical features and the DSFR signature were applied as the candidate predictive factors and tested by the univariate Cox regression analysis to select the factors which were significantly correlated to RFS.

The multivariate Cox regression analysis was performed with the features with a P value < 0.2 in the univariate Cox regression analysis, to identify if the DSFR signature was the independent predictor for RFS. Then, the selected predictive factors in the multivariate Cox regression analysis were used to obtain an integrated nomogram by a stepwise feature selection algorithm. For comparison, a Cox regression model was also built with the DSFR signature alone.

The performance of the constructed nomogram to predict RFS was measured by Harrell’s concordance index (C-index) and time-dependent AUC (tdAUC). The concordance was explored graphically by calibration curves, using the rms Package of R software (version 3.4.4, R Project for Statistical Computing, http://www.r-project.org). Additionally, a decision curve analysis was performed to assess the clinical usefulness and net benefits of the nomogram.

## Data Availability

The data that support the findings of this study are available from the corresponding authors.

## Supplementary Materials

Supplementary Materials Table S1: univariable Cox regression analysis of predictors for ER in the development cohort. Table S2: details of the CT scanners and scan parameters. Table S3: Pearson’s correlation coefficients (R) between the features with the highest weights in the DSFR models based on AP and PP. Table S4: P values of the Pearson correlation analyses between the features with the highest weights in different DSFR models. Figure S1: time-dependent AUC of models in development and validation cohorts. Figure S2: patient recruitment workflow. Figure S3: segmentation network based on classic U-Net architecture. Figure S4: traditional imaging features of CECT by visual analysis.
