# Supplementary material for: Deep Segmentation Feature-Based Radiomics Improves Recurrence Prediction of Hepatocellular Carcinoma
Source: BME Front. 2022 Apr 4;2022:9793716. doi: 10.34133/2022/9793716 (PMC10521680; doi:10.34133/2022/9793716)
Supplement: Supplementary Materials — Table S1: univariable Cox regression analysis of predictors for ER in the development cohort. Table S2: details of the CT scanners and scan parameters. Table S3: Pearson’s correlation coefficients (R) between the features with the highest weights in the DSFR models based on AP and PP. Table S4: P values of the Pearson correlation analyses between the features with the highest weights in different DSFR models. Figure S1: time-dependent AUC of models in development and validation cohorts. Figure S2: patient recruitment workflow. Figure S3: segmentation network based on classic U-Net architecture. Figure S4: traditional imaging features of CECT by visual analysis. [file 9793716.f1.zip › Supplementary Materials.docx]

# Supplementary Materials

## 1. Construction of deep learning-based segmentation model

As shown in Figure S3, we built a segmentation network based on the classic U-Net architecture. The network consists of an encoder and a decoder. The encoder contains three down-sample blocks and a convolution block. The down-sample block is composed of three convolution layers, three rectified linear units (ReLU) layers, two batch normalization layers, and a max-pooling layer. The residual block structure is introduced to enhance the ability of feature extraction. The convolution block is composed of three convolution layers, two ReLU layers, and two batch normalization layers. Correspondingly, the decoder contains three up-sample blocks, a convolution block, and a convolution layer. The up-sample block is composed of four convolution layers, four ReLU layers, three batch normalization layers, a concatenation layer, and an up-sample layer. We also introduce the residual block structure in the up-sample block. The encoder can generate the feature with a size of 512×32×32, containing the semantic segmentation information of the input images. The high-level feature is then inputted into the decoder. Combined with the low-level from the corresponding down-sample blocks, the decoder part yields the final probability map of HCC.

We built the segmentation models based on the respective CT images of the arterial phase and portal phase. We trained all the models in 200 epochs, and the basic learning rate (LR) was 1e-5. After every 50 epochs, the LR was decayed to 1/10. The cross entropy was used as the loss function, and the Adam was adopted as the optimizer. Dice similarity coefficient (DSC) was adopted to evaluate the segmentation of HCC. The optimal segmentation model with the best performance was subsequently implemented in feature extraction. The formula for DSC is:

$$DSC \left( A,B \right)=\frac{2|A\cap B|}{\left| A \right|+|B|}$$

where A and B are the ground true and the segmentation result, respectively.

## 2. Semantic segmentation feature extraction

With the down-sample structure, the encoder can gradually capture rich image information during forward propagation. Therefore, the features generated from the encoder are assumed to have the ability to characterize the input image, and are selected as the extracted features. Global average pooling is applied to transform the feature into size 1×512, which is regarded as the deep semantic segmentation feature of the input image. Based on arterial and portal phase segmentation models, we obtained the respective semantic segmentation features of the two phases.

## 3. Prediction performance of different DSFR models

We built the DSFR models based on AP, PP, and dual-phase (DP, using both AP and PP images) with the average AUC of 0.646, 0.740 and 0.697 in the developmental cohort and 0.594, 0.717 and 0.683 in the validation cohort, respectively. The performance of the DSFR model based on PP was better than that based on the fusion of the two phases.

Further, we explored the correlations between the features from the two phases. Ten features with the highest weights were obtained from the models based on AP and PP respectively. The linear correlation coefficients and the corresponding P-values are shown in Table S3 and Table S4, respectively. The results show that only 30% of the correlation were significant, in which 20% indicate low correlation and 10% show medium association.

**Table S3.** Pearson correlation coefficients (R) between the features with the highest weights in the DSFR models based on AP and PP

| **The feature based on arterial phase** | **The feature based on portal phase** | | | | | | | | | |
| --- | --- | --- | --- | --- | --- | --- | --- | --- | --- | --- |
|  | **1** | **2** | **3** | **4** | **5** | **6** | **7** | **8** | **9** | **10** |
| **1** | -0.04 | 0.01 | 0.00 | -0.05 | 0.07 | -0.05 | -0.04 | 0.11 | 0.04 | -0.07 |
| **2** | 0.15 | 0.39 | 0.31 | -0.07 | 0.62 | 0.06 | -0.01 | -0.15 | -0.19 | 0.27 |
| **3** | -0.14 | 0.41 | 0.34 | -0.15 | 0.26 | -0.15 | -0.04 | -0.21 | -0.34 | 0.04 |
| **4** | -0.57 | 0.17 | 0.15 | -0.12 | -0.27 | 0.06 | -0.05 | -0.04 | -0.18 | 0.02 |
| **5** | 0.23 | 0.10 | 0.06 | 0.11 | 0.44 | -0.01 | 0.16 | 0.02 | 0.03 | 0.14 |
| **6** | 0.18 | 0.26 | 0.19 | -0.03 | 0.51 | 0.20 | 0.03 | -0.03 | -0.05 | 0.34 |
| **7** | 0.11 | -0.07 | -0.25 | 0.04 | 0.21 | 0.21 | 0.10 | 0.32 | 0.27 | 0.23 |
| **8** | 0.16 | 0.01 | -0.07 | 0.10 | 0.17 | -0.02 | 0.10 | 0.00 | 0.14 | -0.02 |
| **9** | 0.00 | 0.01 | 0.08 | 0.01 | -0.02 | 0.03 | -0.01 | -0.01 | -0.04 | 0.06 |
| **10** | 0.07 | -0.08 | -0.27 | 0.22 | 0.10 | 0.05 | 0.26 | 0.21 | 0.28 | 0.09 |

**Table S4.** P-values of the Pearson correlation analyses between the features with the highest weights in different DSFR models

| **The feature based on arterial phase** | **The feature based on portal phase** | | | | | | | | | |
| --- | --- | --- | --- | --- | --- | --- | --- | --- | --- | --- |
|  | **1** | **2** | **3** | **4** | **5** | **6** | **7** | **8** | **9** | **10** |
| **1** | 0.688 | 0.945 | 0.969 | 0.595 | 0.435 | 0.596 | 0.669 | 0.215 | 0.649 | 0.476 |
| **2** | 0.105 | 0.000 | 0.001 | 0.479 | 0.000 | 0.523 | 0.918 | 0.106 | 0.040 | 0.003 |
| **3** | 0.128 | 0.000 | 0.000 | 0.103 | 0.004 | 0.111 | 0.681 | 0.022 | 0.000 | 0.635 |
| **4** | 0.000 | 0.056 | 0.110 | 0.199 | 0.003 | 0.492 | 0.600 | 0.642 | 0.056 | 0.788 |
| **5** | 0.012 | 0.256 | 0.519 | 0.233 | 0.000 | 0.885 | 0.084 | 0.845 | 0.758 | 0.128 |
| **6** | 0.048 | 0.004 | 0.039 | 0.733 | 0.000 | 0.026 | 0.748 | 0.754 | 0.600 | 0.000 |
| **7** | 0.241 | 0.443 | 0.006 | 0.701 | 0.022 | 0.019 | 0.265 | 0.000 | 0.003 | 0.010 |
| **8** | 0.084 | 0.885 | 0.466 | 0.290 | 0.068 | 0.863 | 0.273 | 0.971 | 0.125 | 0.838 |
| **9** | 0.974 | 0.942 | 0.410 | 0.938 | 0.815 | 0.716 | 0.880 | 0.948 | 0.626 | 0.521 |
| **10** | 0.463 | 0.361 | 0.003 | 0.015 | 0.261 | 0.623 | 0.005 | 0.022 | 0.002 | 0.351 |

Table S1. Univariable Cox regression analysis of predictors for ER in the development cohort.

Table S2. Details of the CT scanners and scan parameters.

Table S3. Pearson correlation coefficients (R) between the features with the highest weights in the DSFR models based on AP and PP.

Table S4. P-values of the Pearson correlation analyses between the features with the highest weights in different DSFR models.

Figure S1. Time-dependent AUC of models in development and validation cohorts.

Figure S2. Patient recruitment workflow.

Figure S3. Segmentation network based on classic U-Net architecture.

Figure S4. Traditional imaging features of CECT by visual analysis.
