# Supplementary material for: Deep Segmentation Feature-Based Radiomics Improves Recurrence Prediction of Hepatocellular Carcinoma
Source: BME Front. 2022 Apr 4;2022:9793716. doi: 10.34133/2022/9793716 (PMC10521680; doi:10.34133/2022/9793716)
Supplement: Supplementary Materials — Table S1: univariable Cox regression analysis of predictors for ER in the development cohort. Table S2: details of the CT scanners and scan parameters. Table S3: Pearson’s correlation coefficients (R) between the features with the highest weights in the DSFR models based on AP and PP. Table S4: P values of the Pearson correlation analyses between the features with the highest weights in different DSFR models. Figure S1: time-dependent AUC of models in development and validation cohorts. Figure S2: patient recruitment workflow. Figure S3: segmentation network based on classic U-Net architecture. Figure S4: traditional imaging features of CECT by visual analysis. [file 9793716.f1.zip › Table S1.docx]

Table S1. Univariable Cox regression analysis of predictors for ER in the development cohort

|  | **Hazard Ratio (95% CI)** | **P value** |
| --- | --- | --- |
| **DSFR signature** | 6.676 (3.070, 14.886) | <0.001 |
| **Patient demographics** |  |  |
| Gender |  |  |
| Male | Ref |  |
| Female | 0.533 (0.256, 1.113) | 0.094 |
| Age | 0.992 (0.971, 1.013) | 0.469 |
| **Laboratory parameters** |  |  |
| HBsAg |  |  |
| Negative | Ref |  |
| Positive | 2.716 (0.990, 7.452) | 0.052 |
| HBV-DNA, IU/μL |  |  |
| <100 | Ref |  |
| ≥100 | 0.362 (0.759, 1.271) | 0.362 |
| AFP, μg/L |  |  |
| <400 | Ref |  |
| ≥400 | 2.175 (1.362, 3.475) | 0.001 |
| Child grade |  |  |
| A | Ref |  |
| B | 1.768 (0.555, 5.630) | 0.335 |
| TB | 1.005 (0.996, 1.013) | 0.309 |
| ALB, g/L |  |  |
| ≥35 | Ref |  |
| <35 | 1.668 (0.896, 3.106) | 0.106 |
| ALT | 1.001 (0.998, 1.004) | 0.626 |
| GGT | 1.006 (1.004, 1.008) | <0.001 |

ER, early recurrence; CI, confidence interval; DSFR, deep semantic segmentation feature-based radiomics; Ref, Reference; HBsAg, hepatitis B surface antigen; HBV, hepatitis B virus; AFP, alpha-fetoprotein; TB, total bilirubin;
