# Supplementary material for: Deep Segmentation Feature-Based Radiomics Improves Recurrence Prediction of Hepatocellular Carcinoma
Source: BME Front. 2022 Apr 4;2022:9793716. doi: 10.34133/2022/9793716 (PMC10521680; doi:10.34133/2022/9793716)
Supplement: Supplementary Materials — Table S1: univariable Cox regression analysis of predictors for ER in the development cohort. Table S2: details of the CT scanners and scan parameters. Table S3: Pearson’s correlation coefficients (R) between the features with the highest weights in the DSFR models based on AP and PP. Table S4: P values of the Pearson correlation analyses between the features with the highest weights in different DSFR models. Figure S1: time-dependent AUC of models in development and validation cohorts. Figure S2: patient recruitment workflow. Figure S3: segmentation network based on classic U-Net architecture. Figure S4: traditional imaging features of CECT by visual analysis. [file 9793716.f1.zip › Table S2.docx]

Table S2. Details of the CT scanners and scan parameters

| Parameters | Institution 1 | | | Institution 2 |
| --- | --- | --- | --- | --- |
| CT scanner | Toshiba  Aquilion  VISION | Toshiba  Aquilion  PRIME | Toshiba  Aquilion | Philips iCT |
| Tube voltage, kVp | 120 | 120 | 120 | 120 |
| Tube current, mAs | Automatic | Automatic | 250 | Automatic |
| Rotation time, s | 0.5 | 0.5 | 0.5 | 0.5 |
| Detector collimation, mm | 80×0.5 | 80×0.5 | 64×0.5 | 128×0.625 |
| CM concentration, mgI/mL | Ultravist, 300 | Ultravist, 300 | Ultravist, 300 | Loversol, 320 |
| CM dose, ml/kg | 1.5 | 1.5 | 1.5 | 1.5 |
| Injection flow rate, ml/s | 3 | 3 | 3 | 2.5 |
| AP delay time, s | 40 | 40 | 37 | 37 |
| PP delay time, s | 70 | 70 | 70 | 67 |
| Pixel size | 512×512 | 512×512 | 512×512 | 512×512 |
| Slice thickness mm | 1 | 1 | 1 | 1 |
| Slice interval mm | 0.8 | 0.8 | 0.8 | 1 |
| Kernel | FC18 | FC13 | FC13 | Standard |

CT, computed tomography; CM, contrast material; AP, arterial phase; PP, portal phase
