# Supplementary material for: Deep Segmentation Feature-Based Radiomics Improves Recurrence Prediction of Hepatocellular Carcinoma
Source: BME Front. 2022 Apr 4;2022:9793716. doi: 10.34133/2022/9793716 (PMC10521680; doi:10.34133/2022/9793716)
Supplement: Supplementary Materials — Table S1: univariable Cox regression analysis of predictors for ER in the development cohort. Table S2: details of the CT scanners and scan parameters. Table S3: Pearson’s correlation coefficients (R) between the features with the highest weights in the DSFR models based on AP and PP. Table S4: P values of the Pearson correlation analyses between the features with the highest weights in different DSFR models. Figure S1: time-dependent AUC of models in development and validation cohorts. Figure S2: patient recruitment workflow. Figure S3: segmentation network based on classic U-Net architecture. Figure S4: traditional imaging features of CECT by visual analysis. [file 9793716.f1.zip › Table S3.docx]

**Table S3.** Pearson correlation coefficients (R) between the features with the highest weights in the DSFR models based on AP and PP

| **The feature based on arterial phase** | **The feature based on portal phase** | | | | | | | | | |
| --- | --- | --- | --- | --- | --- | --- | --- | --- | --- | --- |
|  | **1** | **2** | **3** | **4** | **5** | **6** | **7** | **8** | **9** | **10** |
| **1** | -0.04 | 0.01 | 0.00 | -0.05 | 0.07 | -0.05 | -0.04 | 0.11 | 0.04 | -0.07 |
| **2** | 0.15 | 0.39 | 0.31 | -0.07 | 0.62 | 0.06 | -0.01 | -0.15 | -0.19 | 0.27 |
| **3** | -0.14 | 0.41 | 0.34 | -0.15 | 0.26 | -0.15 | -0.04 | -0.21 | -0.34 | 0.04 |
| **4** | -0.57 | 0.17 | 0.15 | -0.12 | -0.27 | 0.06 | -0.05 | -0.04 | -0.18 | 0.02 |
| **5** | 0.23 | 0.10 | 0.06 | 0.11 | 0.44 | -0.01 | 0.16 | 0.02 | 0.03 | 0.14 |
| **6** | 0.18 | 0.26 | 0.19 | -0.03 | 0.51 | 0.20 | 0.03 | -0.03 | -0.05 | 0.34 |
| **7** | 0.11 | -0.07 | -0.25 | 0.04 | 0.21 | 0.21 | 0.10 | 0.32 | 0.27 | 0.23 |
| **8** | 0.16 | 0.01 | -0.07 | 0.10 | 0.17 | -0.02 | 0.10 | 0.00 | 0.14 | -0.02 |
| **9** | 0.00 | 0.01 | 0.08 | 0.01 | -0.02 | 0.03 | -0.01 | -0.01 | -0.04 | 0.06 |
| **10** | 0.07 | -0.08 | -0.27 | 0.22 | 0.10 | 0.05 | 0.26 | 0.21 | 0.28 | 0.09 |
