# Supplementary material for: Deep Segmentation Feature-Based Radiomics Improves Recurrence Prediction of Hepatocellular Carcinoma
Source: BME Front. 2022 Apr 4;2022:9793716. doi: 10.34133/2022/9793716 (PMC10521680; doi:10.34133/2022/9793716)
Supplement: Supplementary Materials — Table S1: univariable Cox regression analysis of predictors for ER in the development cohort. Table S2: details of the CT scanners and scan parameters. Table S3: Pearson’s correlation coefficients (R) between the features with the highest weights in the DSFR models based on AP and PP. Table S4: P values of the Pearson correlation analyses between the features with the highest weights in different DSFR models. Figure S1: time-dependent AUC of models in development and validation cohorts. Figure S2: patient recruitment workflow. Figure S3: segmentation network based on classic U-Net architecture. Figure S4: traditional imaging features of CECT by visual analysis. [file 9793716.f1.zip › Table S4.docx]

**Table S4.** P-values of the Pearson correlation analyses between the features with the highest weights in different DSFR models

| **The feature based on arterial phase** | **The feature based on arterial phase** | | | | | | | | | |
| --- | --- | --- | --- | --- | --- | --- | --- | --- | --- | --- |
|  | **1** | **2** | **3** | **4** | **5** | **6** | **7** | **8** | **9** | **10** |
| **1** | 0.688 | 0.945 | 0.969 | 0.595 | 0.435 | 0.596 | 0.669 | 0.215 | 0.649 | 0.476 |
| **2** | 0.105 | 0.000 | 0.001 | 0.479 | 0.000 | 0.523 | 0.918 | 0.106 | 0.040 | 0.003 |
| **3** | 0.128 | 0.000 | 0.000 | 0.103 | 0.004 | 0.111 | 0.681 | 0.022 | 0.000 | 0.635 |
| **4** | 0.000 | 0.056 | 0.110 | 0.199 | 0.003 | 0.492 | 0.600 | 0.642 | 0.056 | 0.788 |
| **5** | 0.012 | 0.256 | 0.519 | 0.233 | 0.000 | 0.885 | 0.084 | 0.845 | 0.758 | 0.128 |
| **6** | 0.048 | 0.004 | 0.039 | 0.733 | 0.000 | 0.026 | 0.748 | 0.754 | 0.600 | 0.000 |
| **7** | 0.241 | 0.443 | 0.006 | 0.701 | 0.022 | 0.019 | 0.265 | 0.000 | 0.003 | 0.010 |
| **8** | 0.084 | 0.885 | 0.466 | 0.290 | 0.068 | 0.863 | 0.273 | 0.971 | 0.125 | 0.838 |
| **9** | 0.974 | 0.942 | 0.410 | 0.938 | 0.815 | 0.716 | 0.880 | 0.948 | 0.626 | 0.521 |
| **10** | 0.463 | 0.361 | 0.003 | 0.015 | 0.261 | 0.623 | 0.005 | 0.022 | 0.002 | 0.351 |
